# Optimizing Energy/Current Fluctuation of RF-Powered Secure Adiabatic Logic for IoT Devices

**DOI:** 10.3390/s25144419

**Published:** 2025-07-16

**Authors:** Bendito Freitas Ribeiro, Yasuhiro Takahashi

**Affiliations:** 1 Graduate School of Engineering, Gifu University, 1-1 Yanagido, Gifu-shi 501-1193, Japan; 2 Department of Electrical, Electronic and Computer Engineering, Faculty of Engineering, Gifu University, 1-1 Yanagido, Gifu-shi 501-1193, Japan; yasut@gifu-u.ac.jp

**Keywords:** adiabatic logic, peak detector, SEAL-RF, WP-ECRL

## Abstract

The advancement of Internet of Things (IoT) technology has enabled battery-powered devices to be deployed across a wide range of applications; however, it also introduces challenges such as high energy consumption and security vulnerabilities. To address these issues, adiabatic logic circuits offer a promising solution for achieving energy efficiency and enhancing the security of IoT devices. Adiabatic logic circuits are well suited for energy harvesting systems, especially in applications such as sensor nodes, RFID tags, and other IoT implementations. In these systems, the harvested bipolar sinusoidal RF power is directly used as the power supply for the adiabatic logic circuit. However, adiabatic circuits require a peak detector to provide bulk biasing for pMOS transistors. To meet this requirement, a diode-connected MOS transistor-based voltage doubler circuit is used to convert the sinusoidal input into a usable DC signal. In this paper, we propose a novel adiabatic logic design that maintains low power consumption while optimizing energy and current fluctuations across various input transitions. By ensuring uniform and complementary current flow in each transition within the logic circuit’s functional blocks, the design reduces energy variation and enhances resistance against power analysis attacks. Evaluation under different clock frequencies and load capacitances demonstrates that the proposed adiabatic logic circuit exhibits lower fluctuation and improved security, particularly at load capacitances of 50 fF and 100 fF. The results show that the proposed circuit achieves lower power dissipation compared to conventional designs. As an application example, we implemented an ultrasonic transmitter circuit within a LoRaWAN network at the end-node sensor level, which serves as both a communication protocol and system architecture for long-range communication systems.

## 1. Introduction

The Internet of Things (IoT) is an innovation-driven technology that integrates advancements such as 5G, artificial intelligence (AI), near field communication (NFC), Low-Power Wide-Area communication (e.g., LoRa and LoRaWAN). IoT forms a vast sensor network that connects billions of smart devices with users and systems, enabling the collection and sharing of data across various applications. As technology advances, IoT devices are increasingly required to operate with low power consumption, support long-range communication, exhibit low latency, and maintain a compact physical form.

Modern IoT devices are often portable or wearable, which limits their available energy supply. As a results, the development of IoT faces two major challenges: achieving energy autonomy [1,2,3,4,5,6] and ensuring security to protect data confidentiality [3,6]. LoRa communication technology is particularly suitable for low data rate applications in the IoT domain. Using wireless modulation, LoRa allows end-node sensors to communicate over long distances. The associated communication protocol and system architecture are managed by LoRaWAN [7].

Despite higher power consumption being a major challenge in the development of IoT devices, environmental energy harvesting has emerged as a viable alternative. Among the available options, RF energy harvesting stands out for its potential to deliver continuous power wirelessly.

Commercial RF energy harvesters—such as PowerCast, e-peas, Energous WattUp, and Ossia Cota—are already available in the market [8]. These conventional RF harvesters work by converting propagated electromagnetic waves into DC power, which is then used to power IoT devices. This conversion process typically involves a full-wave rectifier, voltage multiplier, and voltage regulator. However, substantial energy losses occur during the rectification and regulation stages [5,9,10]. Recent studies have explored energy storage efficiency in capacitor-based energy harvesting systems, demonstrating methods such as tank capacitor switching to maximize energy utilization during inactive charging periods, which is particularly relevant for low-power IoT applications [11]. While recent research has explored the integration of wireless power transfer (WPT) and mobile edge computing (MEC) to address the energy and computational limitations of IoT nodes in intelligent systems [12], our work focuses on a hardware-level solution through adiabatic logic circuit design to reduce power consumption and enhance security in RF-powered IoT devices [13,14].

In this article, charge-recycling-based AC computing presents a promising approach for overcoming power consumption challenges [2,4]. A particularly effective strategy in RF energy-harvesting circuit design is the use of adiabatic logic circuits [15,16]. Several adiabatic logic techniques have been developed for improved energy efficiency, including Efficient Charge Recovery Logic (ECRL) [9], Pass-Transistor Adiabatic Logic (PAL) [17], Complementary Energy Path Adiabatic Logic (CEPAL) [18], and Secure Adiabatic Logic for RF-powered IoT devices (SEAL-RF) [10].

ECRL has been developed to wirelessly power IoT devices by utilizing peak detectors as bulk bias DC voltage sources and phase shifters to generate multi-phase power-clocks [1,4,5]. In contrast, CEPAL requires both a peak detector and a signal shaper [1,4]. The signal shaper is used to convert the RF-harvested bipolar sinusoidal waveform into a unipolar, sinusoidal-like signal, which is suitable for CEPAL operation. PAL also depends on a signal shaper to eliminate the negative components of the harvested AC signal, ensuring correct logic function [2,4,6]. Meanwhile, SEAL-RF enhances energy efficiency and side-channel attack resistance by reusing the harvested bipolar signal to discharge the output capacitance [10].

In this article, we evaluate suitable peak detector designs for bias-voltage generators for the bulk of pMOS in adiabatic logic circuits. Specifically, we employ a diode-connected MOS transistor-based voltage doubler circuit to rectify the harvested sinusoidal signal. Through uniformization and complementary balancing, the current flow during each input transition is optimized to minimize energy and current fluctuations. The core motivation of this research is to develop an energy efficient and secure adiabatic logic circuit capable of directly utilizing harvested RF signals—thereby addressing both power constraints and security vulnerabilities in IoT devices.

Based on this analysis, we propose a novel adiabatic logic circuit topology that offers lower energy consumption, constant current fluctuation, and improved security features. Preliminary results related to this article have been reported in [15,18]. In this article, we provide a more comprehensive analysis, including transistor switching circuit diagrams for different input pairs, detailed evaluation metrics and their analysis (current and energy traces), as well as a conceptual diagram of the circuit’s application in a smart waste bin detector system, which integrates an RF energy harvester with a LoRaWAN communication system.

## 2. Fundamentals of Adiabatic Logic Circuits

The operating principle of adiabatic logic circuits can be understood by examining the process of charging a capacitor in an RC equivalent circuit, in comparison to conventional CMOS logic. A standard CMOS inverter consists of a pull-up and pull-down network; both are connected to a load capacitance (C).

**Figure 1:**
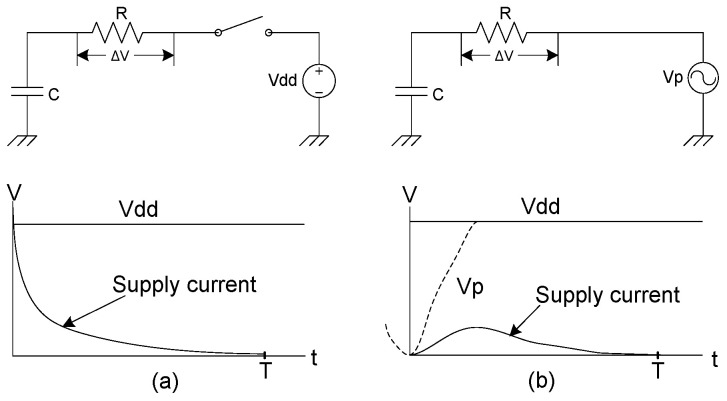
RC equivalent model: (**a**) Conventional CMOS charging and (**b**) Adiabatic charging.

As illustrated in Figure 1a, the load capacitance is connected in series with the MOS transistors of the pull-up or pull-down networks. This configuration can ideally be modeled as a switch in series with a resistor (representing the transistor’s on-resistance).

When the CMOS inverter transitions to a logical high state, a charge of *Q = CV*_dd_ is delivered to the load, and the total energy drawn from the power supply is: *E*_applied_ *= QV*_dd_ *= CV*_dd_^2^. Here, the capacitor *C* is charged from 0 to *V*_dd_, where *V*_dd_ is the DC supply voltage. During this charging process, a significant portion of energy is dissipated across the resistance R in the circuit. The energy dissipation in the resistor can be expressed as the following:

(1) $$\;E_{dissipation}=\frac{1}{2}{{CV}_{dd}}^{2}.$$

This represents the inherent energy loss in conventional CMOS charging, which adiabatic logic aims to minimize by using gradual and reversible charging techniques.

When the CMOS inverter is set to a logical low state, the same amount of energy is dissipated during the discharge process, as no energy can enter the ground rail ($Q\times V_{gnd}=Q\times 0=0$). According to the law of energy conservation, traditional CMOS logic emits heat and wastes energy during the charge and discharge cycles. The total energy dissipated by a CMOS inverter can be calculated by summing the energy used during charging and discharging.

(2) $$\;E_{total}=E_{charge}+E_{discharge}={{CV}_{dd}}^{2}.$$

The power consumption of a CMOS gate operating at a certain frequency f=1/T, where T is the period of the signal, can be calculated as follows:

(3) $$\;P_{total}=\frac{E_{total}}{T}={{CV}_{dd}}^{2}\;f.$$

Adiabatic switching [19] is primarily used to reduce energy consumption during the charging and discharging processes. In adiabatic switching, all nodes are charged with a constant current. This is achieved by supplying an AC power source, allowing the circuit to charge and discharge in a way that enables partial recovery of the supplied energy. Figure 1b illustrates the principle of adiabatic switching, where transitions occur slowly enough to significantly reduce heat dissipation. Accordingly, the energy dissipation across a resistance *R* in adiabatic logic can be derived from the following equation:

(4) $$E_{Adiabatic}=\xi {\left(\frac{{CV}_{dd}}{∆T}\right)}^{2}R∆T\;$$

where *T* is the period of time, and *ξ* is a shape factor that depends on the shape of the clock edges. When the charging period Δ*T* approaches infinity, the dissipated energy theoretically reduces to zero.

## 3. Peak Detector for RF-Powered Adiabatic Logic

### 3.1. RF-Powered Adiabatic Logic Circuit

**Figure 2:**
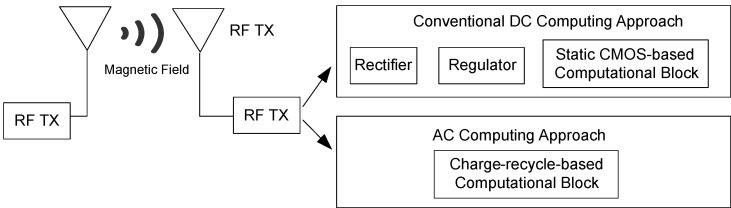
AC computing and conventional approach for RF-powered devices (modified from [1,2,3,4,6]).

The development of wirelessly powered devices conceptually utilizes an AC signal as a power-clock to enable adiabatic charging and energy recycling within the circuit. At the same time, it aims to eliminate energy losses typically incurred during rectification and voltage regulation stages [1,2]. As illustrated in Figure 2, an antenna or coupling coil receives the propagated electromagnetic waves. In conventional systems, a rectification circuit is used to convert the harvested AC signal into a DC signal, followed by a voltage regulator to supply power to the computational block. In the proposed AC computing approach, both the rectifier and voltage regulator are eliminated, thereby reducing overall energy consumption in the circuit.

In the implementation of AC computing methodologies, several fundamental adiabatic logic circuits have been utilized. The WP-ECRL, WP-PAL, and WP-CEPAL circuits represent three proposed approaches for wireless-powered energy harvesting. These methodologies require auxiliary circuitry to support their operation [1,2,3,4,5]. Both WP-ECRL and WP-CEPAL rely on a peak detector for the bulk biasing voltage generator, along with additional phase shifters and signal shapers. In contrast, the WP-PAL circuit requires only a signal shaper.

### 3.2. Peak Detector for RF-Powered Adiabatic Logic

#### 3.2.1. Conventional Peak Detector for RF-Powered Adiabatic Logic

**Figure 3:**
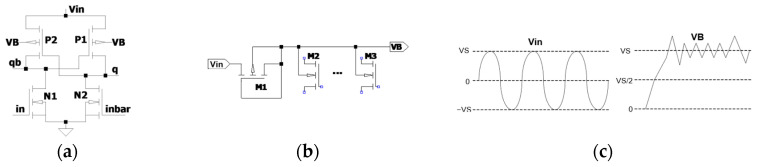
AC power-clock rectification for supplying DC bias to the bulk nodes of pMOS transistors: (**a**) WP-ECRL inverter/buffer circuit, (**b**) conventional peak detector circuit, and (**c**) input voltage and corresponding VB waveform.

The peak detector functions as a rectification stage, supplying a DC voltage to the bulk terminals of the pMOS transistors in the circuit. Figure 3a shows the cross-coupled pMOS transistors used in the WP-ECRL design, which require a stable DC bias at the bulk.

If the bulk terminals are connected directly to an AC signal, as illustrated in Figure 3b,c, the bulk-to-drain junction may become forward biased whenever the junction voltage exceeds the threshold for forward conduction. This results in unwanted power dissipation due to a significant forward bias current through the junction, acting like a diode. To mitigate this issue, a peak detector is employed to supply a DC voltage to the pMOS bulks, effectively preventing forward conduction and reducing power loss.

**Figure 4:**
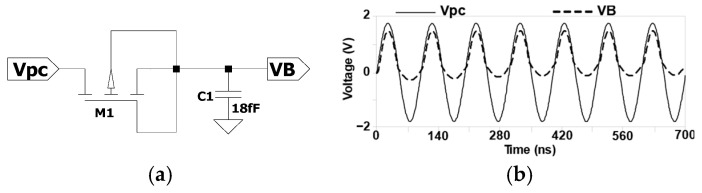
Diode-connected MOS transistor as a conventional rectifier: (**a**) Circuit schematic and (**b**) V_PC_ and VB waveform.

Figure 4 shows a diode-connected pMOS transistor along with the corresponding input and output waveforms used in this study [18]. As depicted in Figure 4b, the output waveform *V*_B_ demonstrates the elimination of the negative voltage component. However, it still does not produce a perfectly constant DC voltage.

#### 3.2.2. Proposed Peak Detector for RF-Powered Adiabatic Logic

Conventional CMOS rectification circuits often employ diode-connected MOS transistors in Dickson charge pump configurations. However, the power efficiency of such rectifiers is limited due to the threshold voltage drop across the switching devices [15,16]. An alternative approach is the Greinacher rectifier, which resembles a two-stage voltage doubler arranged in a bridge configuration.

**Figure 5:**
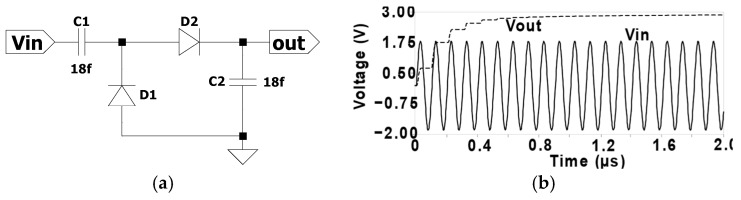
Conventional diode-based voltage doubler rectifier: (**a**) circuit schematic and (**b**) V_in_ and V_out_ waveforms at a power-clock frequency of 10 MHz.

To address these limitations, a new peak detector was designed to provide a DC bias voltage to the bulk terminals of pMOS transistors in RF-powered adiabatic logic circuits [16]. As shown in Figure 5a, the conventional diode-based voltage doubler rectifier uses two diodes and two capacitors for coupling and filtering. Figure 5b illustrates the input and output waveforms at a power-clock frequency of 10 MHz, where the input voltage V_in_ is a bipolar sinusoidal signal, and the output voltage V_out_ is a rectified signal.

**Figure 6:**
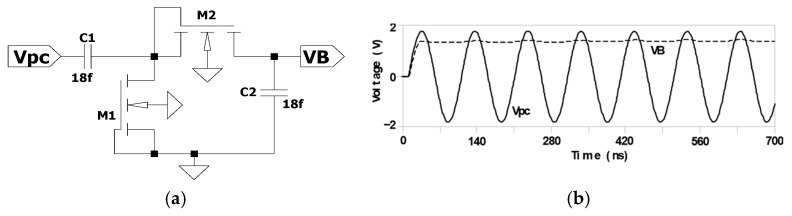
RF-powered rectification for the adiabatic logic circuit: (**a**) circuit schematic and (**b**) *V*_PC_ and *V*_B_ waveforms.

Figure 6a shows a diode-connected nMOS transistor, where the gate and drain are shorted to form a simple voltage divider circuit [16]. This configuration is proposed for RF-powered rectification in adiabatic logic applications. The diode-connected nMOS ensures that the forward-biased MOS transistor operates in saturation, resulting in an output voltage approximately equal to twice the input voltage minus the sum of the threshold voltages of the transistors involved:

(5) $$V_{out}=2V_{in}-V_{th1}-V_{th2}$$

where V_out_ represents V_B_, the voltage applied to the bulk terminal of the transistor; V_in_ refers to the harvested AC signal; and V_th1_ and V_th2_ denote the threshold voltages of the nMOS transistors M1 and M2, respectively.

The CMOS-based voltage doubler helps reduce the voltage drop caused by forward bias and minimizes reverse current leakage. This improves the efficiency of the rectification process, particularly in low-power RF energy harvesting applications. The maximum power conversion efficiency (PCE) of the rectifier can be defined as the following:

(6) $$\;PCE=\frac{P_{out,forward}-P_{leakage}}{P_{input}}$$

where $P_{out,forward}$ refers to the harvested power, $P_{leakage}$ represents the leakage power during the negative half-cycle, and $P_{input}$ refers to the input power.

**Figure 7:**
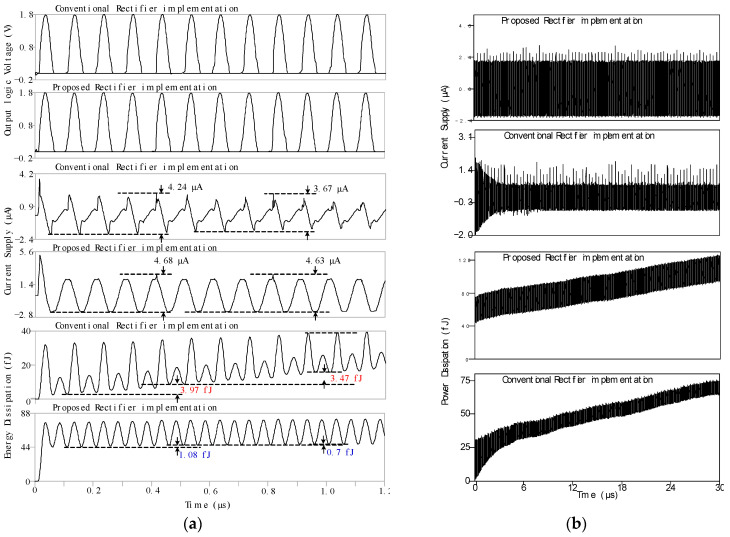
Simulation results of the SEAL-RF NAND/AND adiabatic logic circuit: (**a**) time range of 0–1.2 µs and (**b**) time range of 0–30 µs.

The peak detector for the RF-powered adiabatic logic circuit was evaluated, and the simulation results for the SEAL-RF NAND/AND adiabatic logic circuit are presented in Figure 7. Figure 7a illustrates the first and second clock periods under initial conditions, showing that the proposed peak detector outperforms the conventional design. During these periods, the proposed peak detector exhibits lower energy dissipation and reduced supply current compared to the conventional peak detector. Figure 7b illustrates the simulation results of the SEAL-RF NAND/AND adiabatic logic circuit over a time range of 0–30 µs. The output of the proposed peak detector remained stable throughout the simulation, whereas the conventional peak detector showed instability during the initial period.

## 4. RF-Powered Adiabatic Logic

### 4.1. WP-ECRL Circuit

**Figure 8:**
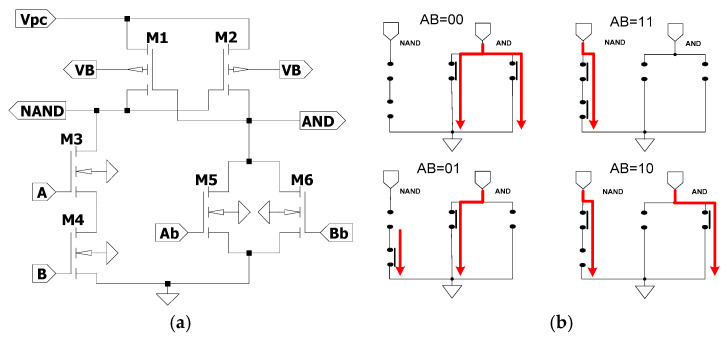
WP-ECRL circuit: (**a**) Circuit schematic and (**b**) switch model equivalent in each transition logic.

WP-ECRL is derived from the ECRL circuit, in which the bulk terminals of the pMOS transistors are connected to a DC power supply. Therefore, the harvested RF signal must be rectified to ensure proper output performance. The WP-ECRL circuit consists of two pMOS transistors (M1 and M2) forming the data block, and four nMOS transistors (M3, M4, M5, and M6) comprising the functional block. In the data block, the bulks of the cross-coupled pMOS transistors are separately connected to a DC voltage *V_B_*, as shown in Figure 8a. Figure 8b illustrates the current paths in an equivalent switch model during different logic transitions. The current paths vary depending on the input transitions, resulting in unbalanced current distribution between the upper and lower branches. For example, during the input transition AB = 00, two long current paths are formed to ground with no short current paths. For the transition AB = 01, one long and one short current path are observed. Compared to other input transitions, these unbalanced current paths can make the circuit more susceptible to power analysis attacks.

### 4.2. SEAL-RF Circuit

**Figure 9:**
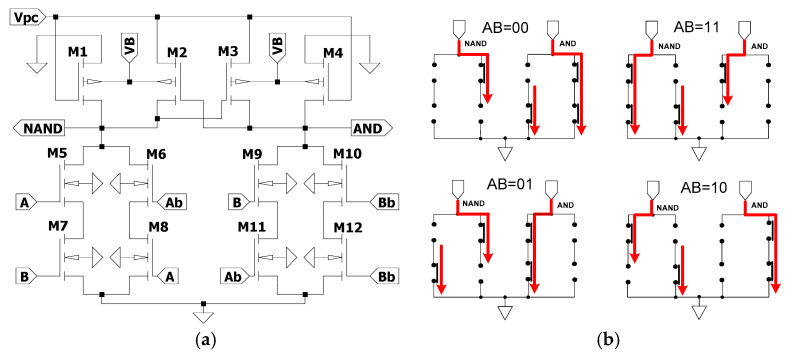
SEAL-RF circuit: (**a**) Circuit schematic and (**b**) switch model equivalent in each transition logic.

The schematic of the RF-powered secure adiabatic NAND/AND logic circuit is shown in Figure 9a. The SEAL-RF NAND/AND circuit consists of four pMOS transistors (M1, M2, M3, and M4), with their bulk terminals connected to the *V*_B_ probe. Among these, pMOS transistors M2 and M3 form the data-hold block, while M1 and M4 function as discharge transistors. Additionally, eight nMOS transistors are included to implement the functional logic block of the adiabatic circuit.

In SEAL-RF, resistance against power analysis attacks is enhanced by reusing the RF-powered bipolar signal to discharge the output capacitances. During each cycle, the output capacitances are discharged, and the negative phase of the power-clock signal is reused for energy recovery. The power-clock signal operates through five distinct phases: **evaluate**, **hold**, **recover**, **discharge**, and **wait**. These phases enable efficient reuse of the wirelessly harvested bipolar signal to discharge the output capacitances, improving both security and energy efficiency [10].

Although the data-hold block enhances resistance against power analysis attacks, the design of the functional block is equally important to achieve improved overall circuit performance. Figure 9b presents the equivalent switch model for various logic transitions. Compared to the WP-ECRL circuit, the SEAL-RF logic circuit exhibits more balanced current paths between the upper and lower branches during different input transitions. For instance, during the input transition AB=00, one long current path flows to ground, and both the upper and lower sides each have one short current path. This more balanced current distribution contributes to a reduced vulnerability to side-channel attacks and an improved energy efficiency.

### 4.3. Optimizing Energy and Current Fluctuations in RF-Powered Secure Adiabatic Logic for IoT Devices

**Figure 10:**
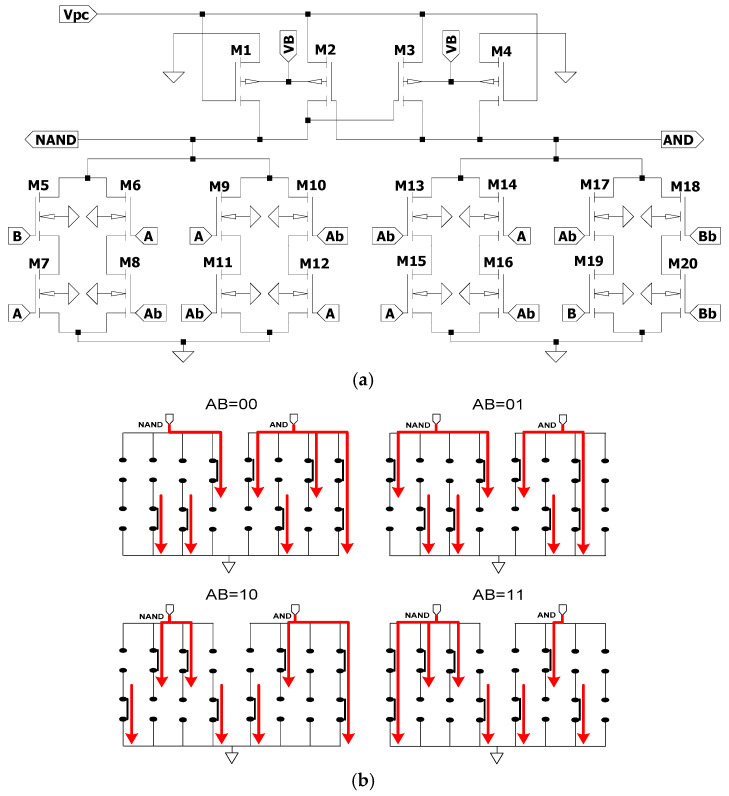
Proposed Circuit: (**a**) Circuit schematic and (**b**) switch model equivalent in each transition logic.

The proposed circuit in this research aims to optimize energy and current fluctuations in RF-powered secure adiabatic logic for IoT devices. As an enhancement to the conventional SEAL-RF architecture, the design improves energy and current performance across various input transitions while also increasing resistance to power analysis attacks. As shown in Figure 10a, the proposed circuit features a cross-coupled pair of pMOS transistors (M2 and M3) serving as the data-hold block, while pMOS transistors M1 and M4 function as discharge transistors. To implement the functional block of the adiabatic logic circuit, a total of 16 nMOS transistors are utilized, enabling improved logic evaluation and energy balancing.

The concept of reducing supply current fluctuations and achieving balanced energy consumption is realized by uniformizing the current flow across each input transition within the function block of the adiabatic logic circuit [16]. The equivalent switch model for each logic transition of the proposed circuit, as shown in Figure 10b, illustrates uniform current flow for every input transition at both the upper and lower sides of the circuit. Specifically, there are three short current paths on the upper side, three on the lower side, and one long current path. For example, during the logic transition AB = 00, three short arrows are present on the upper side, three on the lower side, and one long arrow representing the current flow. This pattern is consistent across all logic transitions, indicating complementary and uniform current distribution. This uniformity significantly contributes to enhancing security by increasing resistance to power analysis attacks.

## 5. Simulation and Results

### 5.1. Simulation Condition

The simulations were conducted using the LTSpice circuit simulator, employing the 0.18 µm standard CMOS process technology which is provided by ROHM Co., Ltd., Kyoto, Japan. The transistor sizes used in the simulation were 0.6 µm width (*W*) and 0.18 µm length (*L*) for both pMOS and nMOS transistors. The power-clock voltage (*V*_PC_) waveform was sinusoidal, with clock frequencies of 1 MHz, 10 MHz, 50 MHz, and 100 MHz. A 1.8 V power-clock was used as the supply voltage throughout the simulations. Output load capacitances for the NAND/AND logic gate was set to 10 fF, 50 fF, 100 fF, 500 fF, and 1000 fF, respectively.

The simulations aimed to evaluate the circuit’s functionality, including input–output voltage behavior, supply current, and power dissipation for both the proposed and conventional circuits. We calculated metrics such as average, standard deviation, normalized energy/current deviation, and normalized standard deviation, based on the maximum and minimum values of energy dissipation and supply current across various input transitions.

The resulting simulation waveforms and summarized calculations provide insight into the circuit’s performance. This analysis enables a comprehensive comparison of energy consumption and supply current waveforms between the proposed and conventional approaches.

### 5.2. Performance Parameters

The parameters presented in this section enable a performance comparison between the proposed circuit and conventional methods, under the assumption that the initial condition is ignored. The evaluation metrics are assessed by quantifying the average energy and current across various input transitions, as well as by calculating the Normalized Energy/Current Deviation (*NED/NCD*) and the Normalized Standard Deviation of Energy/Current (*NSD_E_/NSD_I_*).

The calculations for normalized energy and current deviation are derived from the following equations:

(7) $$\;NED=\frac{E_{max}-E_{min}}{E_{max}}\times 100[\%]$$

(8) $$\;NCD=\frac{I_{max}-I_{min}}{I_{max}}\times 100[\%]$$

(9) $$\;{NSD}_{x}=\frac{\sigma_{x}}{x_{avg}}\times 100\;[\%]$$

(10) $$\;\sigma_{x}=\sqrt{\sum_{i=x_{1}}^{x_{n}}{\left(x_{i}-x_{avg}\right)}^{2}/n[J]}$$

where $\sigma_{x}$ represents the standard deviation of *x*, with *x* referring to *E* when calculating energy fluctuation, and *I* when calculating current fluctuation.

### 5.3. Energy Dissipation and Supply Current Evaluation

#### 5.3.1. Calculation Result Evaluation

The implementation of adiabatic logic circuits aims to reduce both energy consumption and vulnerability to security threats. Table 1 presents a summary of energy dissipation and supply current calculations across various input transitions. The comparison includes three RF-powered adiabatic NAND/AND logic circuits: WP-ECRL, the SEAL-RF circuit, and the proposed circuit designed to optimize energy and current fluctuations.

An analysis of the average energy dissipation, supply current, and corresponding standard deviations for each adiabatic approach reveals that WP-ECRL exhibits the lowest average energy dissipation. However, it also demonstrates significantly higher standard deviations compared to the other methods. Due to the increased number of transistors in the proposed circuit, its average energy (*E*_avg_) and current supply (*I*_avg_) values are slightly higher than those of the SEAL-RF circuit as shown in Table 1. Nevertheless, the proposed design offers a clear advantage in terms of reduced standard deviation.

Overall, under higher load capacitances and across a range of clock frequencies, the proposed circuit consistently achieves lower standard deviations in energy and supply current than both SEAL-RF and WP-ECRL. At lower load capacitances, SEAL-RF exhibits a slightly better standard deviation performance than the proposed circuit.

#### 5.3.2. Simulation Waveform Evaluation

**Figure 11:**
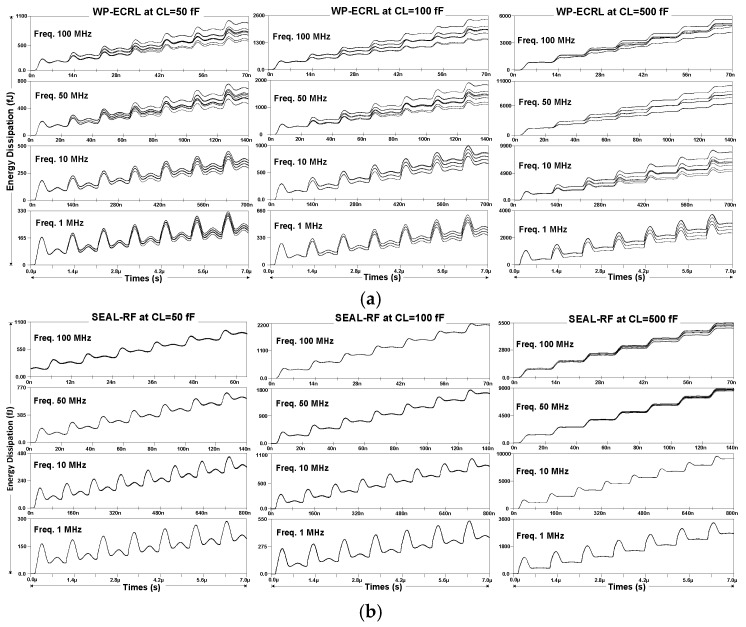

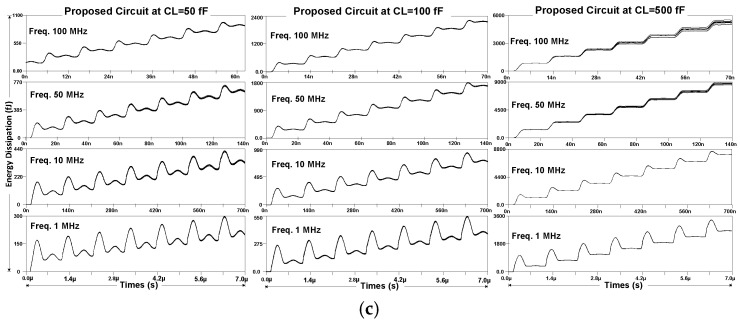
Energy dissipation waveforms from various input transitions: (**a**) WP-ECRL, (**b**) SEAL-RF Circuit, and (**c**) proposed circuit.

The simulation waveforms represent signal shapes plotted as functions of time and magnitude, illustrating energy dissipation, supply current, or timing displacement. Periodic waveforms repeat at regular, constant intervals. Figure 11 shows the energy dissipation waveforms for 16 different input transitions across various clock frequencies for each adiabatic approach.

Figure 11a presents the energy dissipation waveforms of the WP-ECRL circuit at load capacitances of 50 fF, 100 fF, and 500 fF. These waveforms exhibit significant fluctuations across different frequencies and load capacitances compared to those of the SEAL-RF circuit and the proposed circuit.

Figure 11b,c illustrate the energy dissipation waveforms at load capacitances of 50 fF and 100 fF for the SEAL-RF and proposed circuits, respectively. These waveforms show less fluctuation and greater stability compared to other load capacitances. Furthermore, when comparing different clock frequencies applied to the circuits, lower frequencies such as 1 MHz and 10 MHz demonstrate more stable behavior with reduced energy fluctuations across various input transitions, compared to higher frequencies.

**Figure 12:**
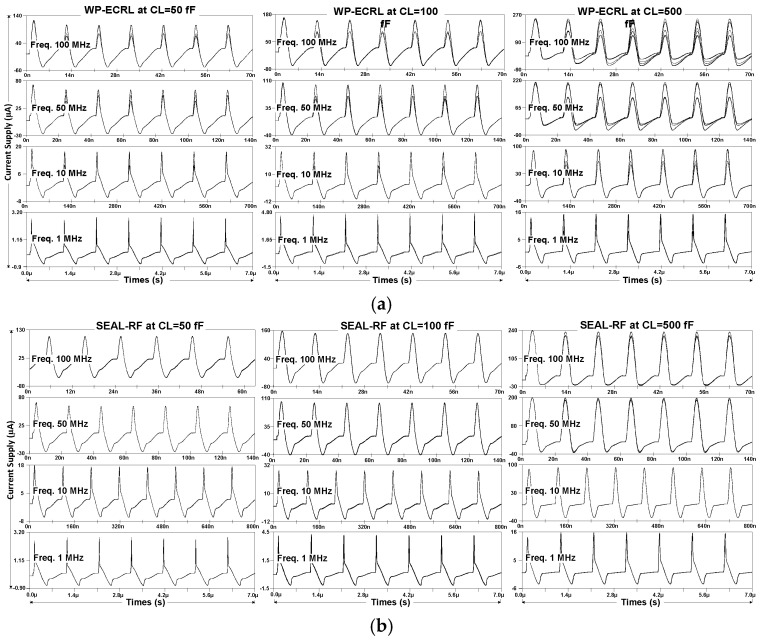

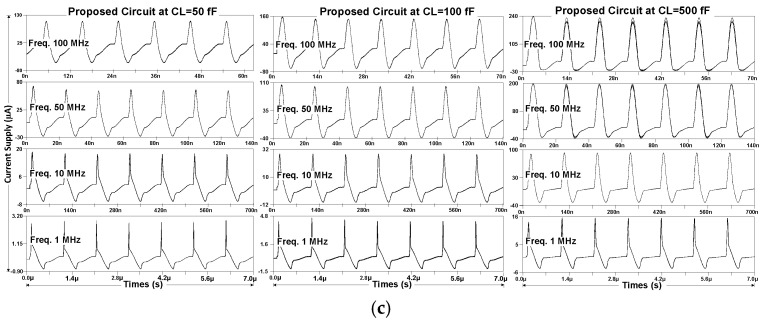
Supply current waveforms from various input transitions (**a**) WP-ECRL, (**b**) SEAL-RF Circuit, and (**c**) proposed circuit.

Figure 12 illustrates the supply current waveforms for various input transitions. Compared to other adiabatic approaches, the WP-ECRL circuit’s supply current waveform in Figure 12a exhibits significant instability and high fluctuation, making it more vulnerable to power attacks. In Figure 12b,c, the SEAL-RF and proposed circuits at load capacitances of 50 fF and 100 fF demonstrate greater stability compared to higher load capacitances. Overall, across all approaches, supply current waveforms at lower load capacitances and various frequencies for the WP-ECRL, SEAL-RF, and proposed circuits show reduced fluctuation and improved stability relative to those at higher load capacitances.

### 5.4. Security Calculation Summary

Besides achieving low power dissipation in VLSI design for IoT devices, addressing security concerns is also a critical challenge. Security metrics, evaluated across various input transitions, load capacitances, and clock frequencies, are summarized in Table 2. The WP-ECRL circuit exhibits the highest percentages in all security parameters, indicating a higher risk and insecurity across different frequencies and load capacitances compared to other adiabatic approaches.

Overall, at higher load capacitances and across various clock frequencies, the proposed circuit demonstrates superior security performance relative to other adiabatic designs. Specifically, at a power-clock frequency of 10 MHz, the proposed circuit shows better normalized energy deviation and normalized standard deviation of energy compared to the SEAL-RF circuit. However, in terms of normalized current deviation and normalized standard deviation of supply current, the SEAL-RF circuit performs better.

**Figure 13:**
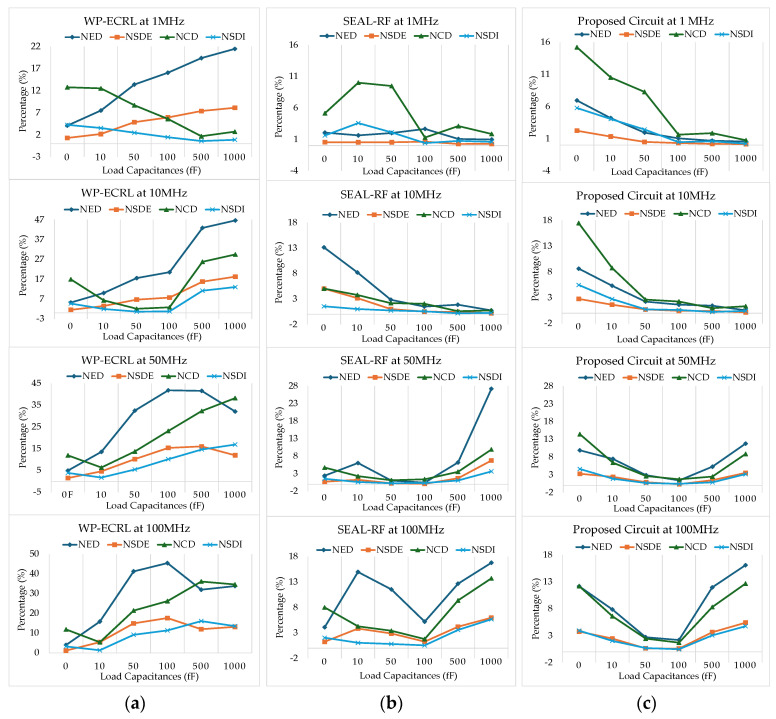
Security evaluation summary graph from various input transitions: (**a**) WP-ECRL circuit, (**b**) SEAL-RF circuit, and (**c**) proposed circuit.

Figure 13 presents summary graphs of the security evaluation across various input transitions. In Figure 13a, the WP-ECRL circuit shows an unstable increase in *NED*/*NCD* and *NSD_E_*/*NSD_I_* as load capacitance increases across different frequencies. This instability compromises the circuit’s security against power analysis attacks. For the SEAL-RF circuit, Figure 13b reveals that at lower frequencies (1 MHz, 10 MHz) and lower load capacitances (0, 10 fF, 50 fF), *NED*/*NCD* and *NSD_E_*/*NSD_I_* are unstable and indicate a higher risk compared to higher load capacitances. Similarly, at higher load capacitances (50 fF, 100 fF, 500 fF) and higher frequency clocks (500 MHz, 1000 MHz), the circuit is also vulnerable to power attacks. However, at a power-clock frequency of 50 MHz and load capacitances of 50 fF and 100 fF, the SEAL-RF circuit demonstrates significantly improved security compared to other conditions. Figure 13c illustrates the security evaluation of the proposed circuit, which shows enhanced security and stability compared to the SEAL-RF circuit, particularly at load capacitances of 50 fF and 100 fF across various clock frequencies.

## 6. Discussion

Conceptually, RF energy harvested from the environment is in the form of a bipolar sinusoidal waveform, which typically requires rectification and regulation to supply a stable DC voltage to conventional computational blocks, as illustrated in Figure 2. In contrast, AC computing approaches, such as adiabatic logic, leverage charge recycling operations to directly utilize harvested signals without the need for rectification and regulation. A key design strategy for this approach involves implementing adiabatic logic circuits. Although a bipolar signal can directly power an adiabatic logic circuit, as shown in Figure 3, a DC voltage is still required at the bulk terminals of the pMOS transistors. To address this requirement, the proposed peak detector circuit in Figure 6 is introduced to enhance the performance of RF-powered adiabatic logic by supplying a stable DC bias.

Several wirelessly powered adiabatic logic circuits have been evaluated in this work, including the WP-ECRL circuit, the SEAL-RF circuit, and the proposed design. The WP-ECRL circuit, a variation of the traditional ECRL, utilizes a bipolar signal as its power-clock, as depicted in Figure 8a. It employs two pMOS transistors as data blocks and four nMOS transistors as functional blocks. However, as shown in Figure 8b, it suffers from unbalanced current flow across various input transitions. To improve upon the limitations of WP-ECRL, the SEAL-RF circuit was introduced. In addition to the data block pMOS transistors, SEAL-RF incorporates two additional pMOS transistors as discharge elements. This design enables the harvested RF bipolar signal to discharge the output capacitance effectively, improving energy efficiency and resistance to power analysis attacks. Figure 9a shows that SEAL-RF uses eight nMOS transistors in its functional block, while Figure 9b demonstrates improved balance in current distribution across input transitions.

The proposed circuit further enhances the functional block of the SEAL-RF design. As shown in Figure 10a, it integrates 16 nMOS transistors to optimize energy and current fluctuations. The switch-level model in Figure 10b confirms the presence of uniform and complementary current paths across input transitions, both at the upper and lower branches of the circuit, contributing to improved security and power stability.

Energy dissipation and supply current calculation summaries for various input transitions are presented in Table 1. Among the evaluated circuits, the WP-ECRL exhibits the lowest average energy dissipation and supply current values compared to the SEAL-RF and the proposed circuit. This is primarily attributed to the smaller number of transistors utilized in both its data and functional blocks. However, the standard deviation values for the WP-ECRL circuit—across different load capacitances and clock frequencies—are generally higher than those observed in the other approaches.

In contrast, the proposed circuit demonstrates significantly lower standard deviations across various input transitions, indicating improved stability. The only exception occurs at the 10 fF load capacitance, where the standard deviation remains relatively high.

Energy dissipation waveforms for various input transitions are shown in Figure 11. Both the SEAL-RF and proposed circuits exhibit reduced energy fluctuations and improved security when compared to the WP-ECRL circuit. Nevertheless, at a power-clock frequency of 50 MHz and 100 MHz with a 500 fF load capacitance, both circuits show increased energy fluctuation. Similarly, as shown in Figure 12, the supply current waveforms at 500 fF demonstrate noticeable fluctuations at 50 MHz and 100 MHz.

Overall, the SEAL-RF and proposed circuits perform more securely and with lower fluctuation at load capacitances of 50 fF and 100 fF. Table 2 and Figure 13 provide a summary of the security evaluation across various input transitions, load capacitances, and clock frequencies.

In summary, comprehensive analysis indicates that the proposed circuit achieves the best performance at load capacitances of 50 fF and 100 fF under various frequency conditions. These capacitance values are associated with minimal energy and current fluctuations, improved security, and enhanced resistance to power analysis attacks.

## 7. Application Example of the Ultrasonic Transmitter Circuit

LoRaWAN is an unlicensed Low-Power Wide-Area (LPWA) network designed to support large-scale IoT deployments. It enables long-range communication with minimal power consumption and can support the integration of adiabatic logic-based circuits for wireless energy harvesting applications.

A typical LoRaWAN architecture comprises end nodes (e.g., LoRa-enabled sensor nodes), gateways, a network server, and an application server. To facilitate data flow between the end nodes and the gateway, an intermediate device called the Input–Output (I/O) Controller may be employed for data management and relay.

As a practical implementation of the proposed RF-powered adiabatic logic circuit, an ultrasonic sensor system is used to detect the presence or level of objects—such as waste in a bin. The sensor’s output is processed through a 10-bit CD4017B counter, then transmitted to the I/O Controller, which forwards the data via the LoRaWAN network.

**Figure 14:**
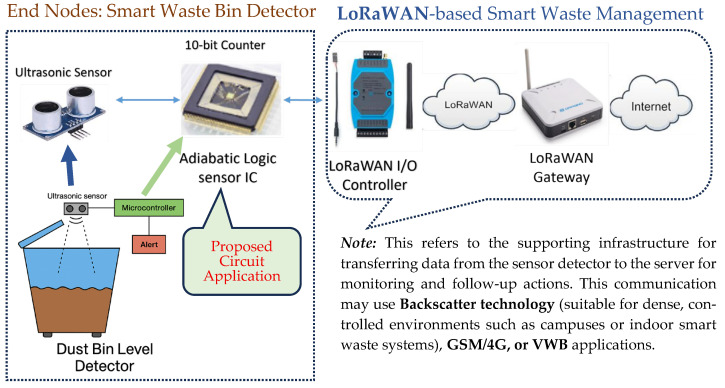
A concept design of the proposed adiabatic energy harvesting sensor network system in LoRaWAN technology.

The overall concept of this energy harvesting sensor network is illustrated in Figure 14. In the design, the ultrasonic sensor emits pulses and measures the time delay of reflections to estimate the waste level. When the detected distance falls below a predefined threshold (indicating a full bin), a microcontroller processes the data and triggers an alert.

This microcontroller is driven by the adiabatic logic circuit, which constitutes a central contribution of this research, offering enhanced energy efficiency at the sensor node level.

For wireless communication, the LoRaWAN I/O Controller collects the processed sensor data and transmits it to the LoRaWAN Gateway, which then sends it to the Internet via suitable backhaul (e.g., GSM/4G or Ethernet). The cloud infrastructure ultimately forwards the data to remote monitoring platforms for analysis, alerts, and follow-up actions.

The CD4017BC is a 10-stage Johnson counter with 10 decoded outputs and a carry-out bit. It includes a reset input that clears the counter to zero when driven by a logical “1”. The counter advances on the rising edge of the clock signal, provided that the clock enable input is held low (“0”). With each rising clock pulse, the counter increments by one, and upon reaching the ninth output, it resets to zero on the next pulse, thereby operating in a cyclic manner.

In this work, we evaluated the energy dissipation of the conventional CMOS and proposed RF-powered adiabatic CD4017B counter. Power dissipation for both versions was analyzed using LTSpice simulation. As shown in Table 3, the dynamic energy dissipation of the adiabatic implementation is 216 pJ per cycle, while the conventional CMOS version (operating at 3.3V) dissipates 3.75 mJ per cycle. These results clearly demonstrate that the proposed adiabatic logic circuit significantly reduces power consumption compared to traditional CMOS designs.

Table 4 compares CMOS circuit designs from 2016 to 2023 with the proposed work for wireless sensor applications. The proposed design uses 0.18 µm CMOS technology with a ±1.8 V sine wave supply and achieves a power consumption of 216 pJ/cycle. This work targets a counter circuit application and remains at the simulation level without physical fabrication. Among prior works, CMOS technologies range from 0.5 µm to 65 nm, with various supply voltages such as fixed DC levels or sine waves. Power consumption varies widely, from 128 fJ in 2016 [20] to 839 pJ in 2023 [10]. Application types include logic functions, clock data recovery (CDR), and stepwise gate drivers, while operating frequencies range from 10 MHz up to 80 MHz or higher. The proposed work demonstrates a balance of modern technology scaling, moderate frequency operation (<100 MHz), and energy efficiency, suitable for secure and low-power counter-based applications.

## 8. Conclusions

In this work, a novel RF-powered adiabatic logic circuit has been proposed to optimize energy and current fluctuations for secure IoT applications. The proposed circuit enhances the existing SEAL-RF design by further optimizing its functional block, integrating 16 nMOS transistors to create more uniform and complementary current paths. As a result, the circuit achieves improved stability, lower energy and current fluctuations, and an enhanced resistance to power analysis attacks when compared to both the SEAL-RF and WP-ECRL circuits.

Comprehensive simulations were conducted across various clock frequencies (1 MHz, 10 MHz, 50 MHz, and 100 MHz) and load capacitances. The results show that the proposed design performs most efficiently and securely at load capacitances of 50 fF and 100 fF, exhibiting minimal fluctuations and superior energy stability.

Furthermore, the applicability of the proposed circuit within a LoRaWAN network—particularly for low-power end-node sensor applications—demonstrates its practical advantage in minimizing power dissipation compared to conventional CMOS approaches. The present work exhibits competitive performance in both energy efficiency and operational frequency, while utilizing a mature and cost effective 0.18 μm CMOS technology node. These results position the proposed circuit as a promising candidate for secure, energy-efficient adiabatic logic implementations in wirelessly powered IoT systems.

## Figures and Tables

**Table 1:** Energy dissipation and supply current calculation summary from various input transitions.

		WP-ECRL					SEAL-RF					Proposed				
CL (F)		10f	50f	100f	500f	1000f	10f	50f	100f	500f	1000f	10f	50f	100f	500f	1000f
1 MHz	E_avg_ (fJ)	61.89	194.1	352.6	2238.9	5296.8	62.22	186.1	361.8	2224.8	5373.2	65.17	189.8	366.0	2230.5	5389.0
	σE (fJ)	1.36	9.46	22.35	165.7	431.1	0.33	0.98	2.25	5.69	14.56	0.86	0.94	1.12	4.12	8.51
	I_avg_ (µA)	0.96	3.27	5.36	19.52	34.25	1.00	3.27	5.22	19.54	34.57	1.07	3.33	5.29	19.75	34.67
	σI (µA)	0.03	0.08	0.08	0.12	0.30	0.04	0.07	0.02	0.15	0.20	0.04	0.08	0.02	0.12	0.07
10 MHz	E_avg_ (fJ)	68.02	254.7	561.2	4195.0	10,115.9	79.12	267.0	583.3	5028.6	12,262.8	72.56	260.5	577.9	5043.3	12,308.1
	σE (fJ)	2.36	17.0	43.4	660.3	1846.1	2.52	2.70	3.07	22.88	26.10	1.19	1.88	2.66	21.09	20.87
	I_avg_ (µA)	9.08	23.01	37.16	116.3	175.5	11.30	24.95	38.04	124.3	185.6	10.05	23.60	37.93	124.8	185.6
	σI (µA)	0.18	0.15	0.28	13.02	22.84	0.12	0.19	0.22	0.25	0.50	0.28	0.17	0.24	0.30	0.82
50 MHz	E_avg_ (fJ)	79.53	371.3	870.8	4386.2	5289.2	81.59	416.9	1042.6	4917.4	5692.4	87.35	427.1	1055.9	4960.9	5822.7
	σE (fJ)	3.65	37.94	133.9	704.5	633.9	1.07	1.17	1.46	85.28	385.9	2.10	3.85	3.86	73.84	205.9
	I_avg_ (µA)	38.12	84.85	129.1	243.5	254.3	39.72	88.69	137.2	226.6	235.1	41.65	90.12	138.0	227.3	235.7
	σI (µA)	0.66	4.64	13.13	35.68	43.05	0.26	0.28	0.54	2.49	8.65	0.79	0.61	0.67	1.97	7.49
100 MHz	E_avg_ (fJ)	89.79	457.7	1042.8	2654.4	2879.6	94.34	547.6	1252.2	2880.7	3141.7	100.9	557.8	1274.5	2918.1	3178.6
	σE (fJ)	4.86	68.31	183.7	317.6	379.2	3.64	15.80	15.96	121.5	187.9	2.47	3.56	7.58	106.0	171.9
	I_avg_ (µA)	70.24	144.7	201.0	266.8	264.4	72.57	153.0	210.5	247.6	256.8	75.76	155.0	211.3	248.9	260.9
	σI (µA)	0.92	13.27	22.88	42.94	35.83	0.77	1.27	1.17	8.86	14.59	1.54	1.08	0.99	7.59	12.41

Note: In the standard deviation (σ) rows, blue-colored numbers in the table indicate lower values when comparing the proposed circuit to other conventional adiabatic circuits, while red-colored numbers indicate higher values.

**Table 2:** Security calculation summary from various input transitions.

		WP-ECRL					SEAL-RF					Proposed Circuit				
CL (F)		10f	50f	100f	500f	1000f	10f	50f	100f	500f	1000f	10f	50f	100f	500f	1000f
1 MHz	NED (%)	7.52	13.40	16.07	19.38	21.47	1.63	2.00	2.65	1.05	0.97	4.19	1.93	1.04	0.67	0.55
	NSD_E_ (%)	2.19	4.87	5.94	7.40	8.14	0.53	0.53	0.62	0.26	0.27	1.32	0.50	0.31	0.18	0.16
	NCD (%)	12.52	8.70	5.61	1.68	2.72	10.00	9.48	1.28	3.11	1.85	10.51	8.26	1.62	1.85	0.75
	NSD_I_ (%)	3.55	2.49	1.47	0.61	0.89	3.58	2.10	0.40	0.75	0.59	4.05	2.41	0.46	0.59	0.21
10 MHz	NED (%)	10.04	17.53	20.51	42.85	46.63	8.22	2.85	1.52	1.88	0.76	5.32	2.25	1.65	1.45	0.55
	NSD_E_ (%)	3.47	6.67	7.73	15.74	18.25	3.18	1.01	0.53	0.45	0.21	1.64	0.72	0.46	0.42	0.17
	NCD (%)	6.29	2.06	2.80	25.78	29.48	3.80	2.19	2.08	0.61	0.75	8.77	2.63	2.27	0.96	1.34
	NSD_I_ (%)	1.95	0.64	0.75	11.19	13.01	1.03	0.74	0.58	0.20	0.27	2.75	0.74	0.62	0.24	0.44
50 MHz	NED (%)	13.56	32.50	41.84	41.58	32.10	6.07	1.08	0.47	6.23	27.19	7.49	2.89	1.41	5.30	11.81
	NSD_E_ (%)	4.59	10.22	15.38	16.06	11.98	1.31	0.28	0.14	1.73	6.78	2.41	0.90	0.37	1.49	3.54
	NCD (%)	6.36	13.72	23.15	32.33	38.26	2.32	1.15	1.44	3.56	9.91	6.43	2.63	1.75	2.51	8.89
	NSD_I_ (%)	1.74	5.46	10.17	14.65	16.93	0.66	0.32	0.39	1.10	3.68	1.90	0.68	0.48	0.87	3.18
100 MHz	NED (%)	15.84	41.24	45.46	31.99	33.84	14.98	11.55	5.21	12.67	16.79	7.87	2.71	2.19	11.98	16.10
	NSD_E_ (%)	5.41	14.93	17.62	11.97	13.17	3.86	2.89	1.27	4.22	5.98	2.45	0.64	0.59	3.63	5.41
	NCD (%)	5.39	21.42	26.20	36.13	34.62	4.29	3.42	1.79	9.39	13.72	6.62	2.45	1.70	8.32	12.68
	NSD_I_ (%)	1.31	9.17	11.39	16.09	13.55	1.06	0.83	0.56	3.58	5.68	2.03	0.69	0.47	3.05	4.76

Note: In the standard deviation (σ) rows, blue-colored numbers in the table indicate lower values when comparing the proposed circuit to other conventional adiabatic circuits, while red-colored numbers indicate higher values.

**Table 3:** Static Power Dissipation Comparison through LTSpice Simulation.

Conventional CD4017B	Proposed Adiabatic Logic Implementation
3.75 mJ/cycle (3.3V CMOS)	216 pJ/cycle

**Table 4:** Comparison of Similar CMOS circuits across various works with the proposed work.

Year	2016 [20]	2018 [21]	2021 [22]	2022 [23]	2023 [10]	This Work
CMOS Technology	0.18 μm	0.5 μm	90 nm	65 nm	0.6 μm	0.18 μm
Supply Voltage	0.25 V	0.3–0.5 V	0.6 V	−1.8–1.8 V sine wave	0.05 V	−1.8–1.8 V sine wave
Power (J/Cycle)	128 f	-	(12.9 nW)	-	839 p	216 p
Fabrication	N (Sim. only)	Y	Y	N (Sim. only)	Y	N (Sim. only)
Application Level	256 Inv-chain	Logic	CDR	Logic	Stepwise gate driver	Counter circuit
Operating Freq. (Hz)	80M	10-10 M	-	13.56 M	500	<100 M

## Data Availability

Data is available on request from the authors.
